# Visit-to-Visit Heart Rate Variability Is Positively Associated With the Risk of Adverse Cardiovascular Outcomes

**DOI:** 10.3389/fcvm.2022.850223

**Published:** 2022-03-07

**Authors:** Rong Zeng, Zuoguang Wang, Wenli Cheng, Kun Yang

**Affiliations:** ^1^Centre of Hypertension, Beijing Anzhen Hospital, Capital Medical University, Beijing, China; ^2^Beijing Institute of Heart, Lung and Blood Vessel Diseases, Beijing Anzhen Hospital, Capital Medical University, Beijing, China; ^3^Cardiac Care Unit, Department of Cardiology, Beijing Anzhen Hospital, Capital Medical University, Beijing, China

**Keywords:** visit-to-visit heart rate variability (VVHRV), major adverse cardiovascular events (MACEs), intensive blood pressure control, Systolic Blood Pressure Intervention Trial (SPRINT), hypertension

## Abstract

**Introduction:**

Previous studies found visit-to-visit heart rate variability (VVHRV) may be positively associated with risks of several cardiovascular events, but whether VVHRV affected the benefit of intensive blood pressure control remained unknown. In this study, we assessed the risk of the composite cardiovascular outcomes associated with VVHRV among the older patients with hypertension and evaluated whether the benefit of intensive blood pressure control in the prevention of the composite cardiovascular outcomes was consistent in the context of elevated VVHRV.

**Methods:**

This was a *post-hoc* analysis of the Systolic Blood Pressure Intervention Trial (SPRINT). We explored the relationship between VVHRV and the composite cardiovascular outcomes by multivariate Cox proportional hazard regressions. The primary endpoint was the composite cardiovascular outcomes, same as SPRINT, defined as a composite of myocardial infarction, stroke, heart failure, and/or death from cardiovascular causes. We used multiple adjustment models for all regressions.

**Results:**

Nine thousand two hundred and fourty-seven patients from the SPRINT were included in our analysis. We found a positive association between VVHRV and the risk of composite cardiovascular outcomes among the elderly with hypertension. Per 1 CV increment in HRCV, the hazard ratio of the risk of composite cardiovascular outcomes was 1.04 (95CI: 1.03, 1.05) in the fully adjusted Model. The benefit of intensive blood pressure control in managing cardiovascular events was consistent in different VVHRV subgroups. There was no significant interaction in other confounders.

**Conclusion:**

We found the VVHRV was associated with the composite cardiovascular outcomes among the elderly with hypertension, intensive blood pressure control did not change the above association, and the benefits of intensive blood pressure management were consistent across different VVHRV groups.

## Introduction

High resting heart rate (RHR) was reported as a predictor of cardiovascular diseases and cardiovascular mortality in the general population as well as in the elderly according to several epidemiology studies (1). In patients with hypertension, a positive association between RHR and cardiovascular mortality was also revealed early in 1993 in the Framingham Study (2). However, using random observations to estimate the average level of heart rate may bring measurement errors, as the measurements fluctuated unpredictably around their true values (3).

In order to solve the measurement error of RHR, the coefficient of variation of heart rate (HRCV) was often calculated to represent the visit-to-visit heart rate variability (VVHRV), which became an emerging risk indicator for cardiovascular diseases. Accumulating evidence proved that VVHRV may be positively associated with risks of several cardiovascular events, such as myocardial infarction, heart failure, atrial fibrillation, and cardiovascular mortality (4–7). The Systolic Blood Pressure Intervention Trial (SPRINT) trial showed that intensive blood pressure control showed significant better cardiovascular outcomes, after which the American hypertension guideline quickly reduced the blood pressure target (8, 9). Sobieraj et al. reported a more potent increase in cardiovascular risk associated with higher RHR in the intensive blood pressure treatment than in the standard treatment group (10). However, whether long-term RHR variability affected the benefit of intensive blood pressure control remained unknown. Therefore, in this study, we aim to assess the risk of the composite cardiovascular outcomes associated with long-term RHR variability among the older patients with hypertension and to evaluate whether the benefit of intensive blood pressure control in prevention of the composite cardiovascular outcomes was consistent on condition of elevated long-term RHR variability.

## Methods

### Data Source and Study Population

We performed a secondary analysis of the SPRINT trial. Data were obtained from the National Institutes of Health Biologic Specimen and Data Repository Information Coordinating Center (https://biolincc.nhlbi.nih.gov/studies/sprint/). The rationale, protocols, and main results of the SPRINT have been published previously (8, 11). The SPRINT trial was conducted in 102 clinical sites in the United States and enrolled 9,361 participants, all of whom were randomly assigned to either the intensive blood pressure control group (systolic blood pressure <120 mmHg) or standard blood pressure control group (systolic blood pressure <140 mmHg). We restricted the analysis to participants with baseline heart rate and at least two follow-up heart rate records available. 9,247 participants were included in this study, while 17 patients with no baseline heart rate and 97 with <2 follow-up heart records were excluded from this analysis.

### Visit-to-Visit Heart Rate Variability and Outcomes

We used coefficient of variation for all heart rate records (HRCV) to assess the visit-to-visit heart rate variability (VVHRV). HRCV was calculated using the following formula: HRSD = $\sqrt{{\sum \left(HR_{i}-HR_{mean}\right)}^{2}/\left(n-1\right)}$; HRCV = HRSD/HR_mean;_ where HR_i_ was the heart rate record at each visit and HR_mean_ was the mean of all heart rate records. All participants included in this study were grouped into 3 tertiles according to HRCV.

The primary outcome of this study was the composite cardiovascular outcomes. The composite cardiovascular outcomes were the first occurrence of cardiovascular events after randomization, including myocardial infarction (MI), non-MI acute coronary syndrome (non-MI ACS), new-onset stroke, heart failure, and death attributable to CVD. The definition of clinical outcomes was previously published in the SPRINT protocol (8).

### Statistical Analysis

We assessed baseline characteristics and crude outcomes stratified by the tertiles of HRCV: T1: the low tertile; T2: the middle tertile; T3: the high tertile. Categorical variables were expressed as frequencies and percentages. Means ± standard deviations or medians (interquartile ranges) were used for continuous variables based on the distribution of data. Differences in categorical variables were evaluated using the Chi-square analysis. The two-tailed *t*-test (normal distribution) or Mann-Whitney U test (skewed distribution) were used to determine any significant differences between the means or medians of the groups. The normal distribution of data was assessed by Kolmogorov-Smirnov test.

According to the recommendation of the Strengthening the Reporting of Observational studies in Epidemiology (STROBE) statement (12). we constructed unadjusted, minimally adjusted, and fully adjusted cox models simultaneously to assess the association between HRCV tertiles and the composite cardiovascular outcomes. The variables with baseline difference and variables that might influence the outcome were included as covariates. Model 1 was adjusted for none; Model 2 was adjusted for age, sex, race, and treatment arms; and Model 3 was further adjusted for age, sex, race, treatment arms, baseline systolic blood pressure, baseline heart rate, smoking status, eGFR, serum creatinine, urine albumin/creatinine ratio, Framingham 10-year CVD risk score, previous CVD, previous chronic kidney disease. We used the graphical methods via the scaled Schoenfeld residuals to examine the proportional hazard assumption. All models met the proportional hazard assumption. The relationship between HRCV as a continuous variable and outcomes according to treatment arms was also evaluated using the three models above. The continuous relationship between VVHRV and outcomes (Model 3) in various subgroups (age, gender, previous CVD, previous chronic kidney disease, heart rate categories, systolic blood pressure categories, Framingham 10-year CVD risk) were also evaluated by stratified analyses and interaction tests. The dose-response relationship between HRCV and outcomes (Model 3) was conducted using generalized additive model (GAM) and fitting smooth curve (restricted cubic splines) with four knots at the 5th, 35th, 65th, and 95th percentiles. To determine whether the benefits of intensive blood pressure control remain robust in different HRCV tertiles, we further perform interaction analyses and stratified analyses.

All analyses were performed using the statistical software packages R (The R Foundation; http://www.R-project.org). Statistical significance was set at *P* < 0.05.

## Results

**Figure 1:**
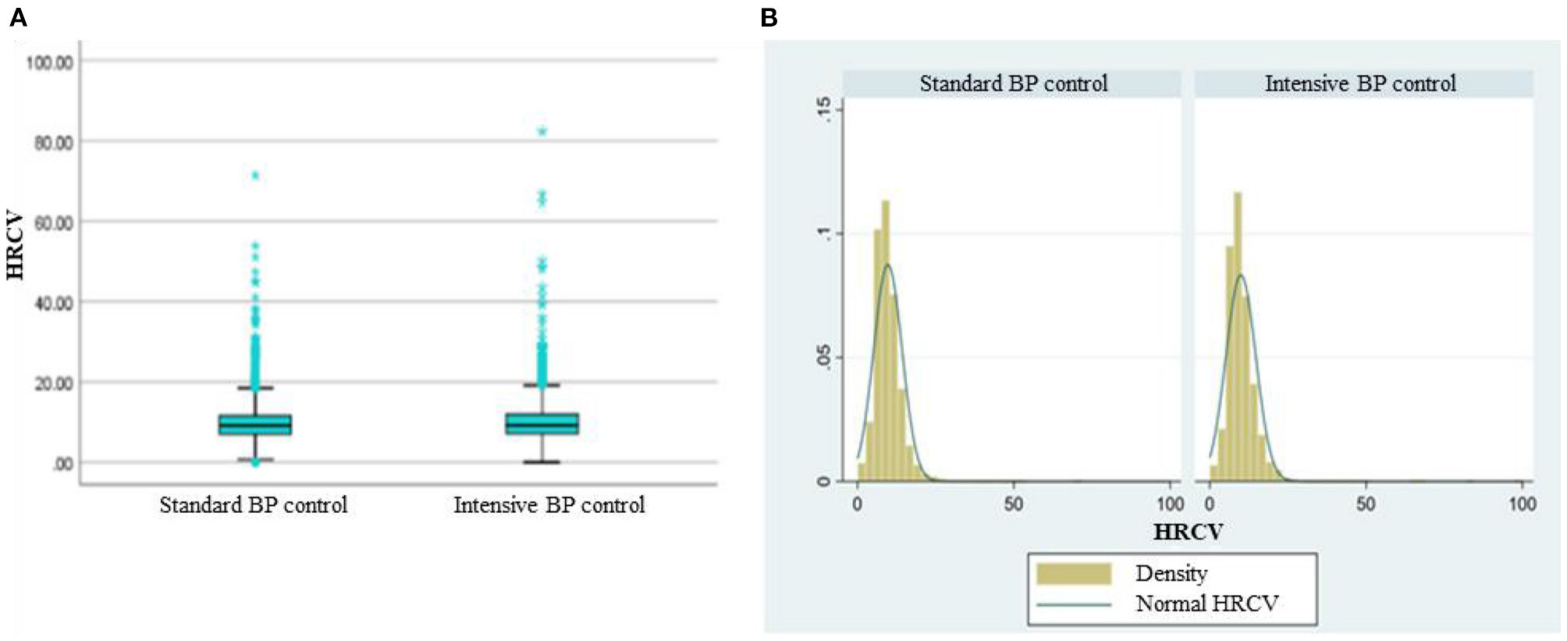
The distribution of HRCV according to treatment arms. **(A)** Box plot of HRCV grouped by standard and intensive BP control. No significant difference between the two groups by Mann-whitney *U-*test (*P* = 0.052). **(B)** Density curve of HRCV grouped by standard and intensive BP control. Normal HRCV: normal distribution curve of HRCV.

Baseline characteristics of 9,247 SPRINT participants included in analysis were shown according to the tertiles of HRCV in Table 1. The median HRCV was 9.27 for participants randomly assigned to intensive BP control and 9.07 for those assigned to standard BP control. There was no significant difference in HRCV between the treatment arms (Figure 1A). The density curve in Figure 1B showed a similar distribution of HRCV between the intensive and standard BP control. Compared to those with low HRCV, the participants with higher HRCV seemed to have higher systolic blood pressure, serum creatinine, urine albumin/creatinine ratio, male ratio and lower estimated GFR. The level of total cholesterol, triglycerides, fasting glucose had no significant difference between HRCV tertiles. There was no difference between HRCV tertiles in aged 75 years and older, statin use, and aspirin use. Participants with the high HRCV tertile were more likely to smoke and had a higher Framingham 10-y CVD risk.

**Table 1:** Baseline characteristics and crude outcomes of the participants according to coefficient of variation in resting heart rate.

**Variable**	**Tertiles of CV in resting heart rate**			***P-*value**
	**T1**	**T2**	**T3**	
CV of RHR, median (min–max)	6.34 (0.00–7.76)	9.16 (7.77–10.71)	13.11 (10.71–82.27)	-
*N*	3082	3082	3083	-
Treatment				
Intensive, *n* (%)	1492 (48.41%)	1568 (50.88%)	1573 (51.02%)	0.070
BMI(Kg/m^2^), median (Q1–Q3)	28.94 (25.81–32.51)	28.92 (25.94–33.09)	29.19 (25.99–33.12)	0.059
Age, y				
Overall	68.28 ± 9.28	67.44 ± 9.42	68.01 ± 9.50	0.001
≥75y, *n* (%)	902 (29.27%)	824 (26.74%)	888 (28.80%)	0.063
Sex, *n* (%)				<0.001
Male	1850 (60.03%)	2018 (65.48%)	2098 (68.05%)	
Female	1232 (39.97%)	1064 (34.52%)	985 (31.95%)	
Race, *n* (%)				<0.001
Non-Hispanic White	1863 (60.45%)	1754 (56.91%)	1733 (56.21%)	
Non-Hispanic Black	827 (26.83%)	941 (30.53%)	984 (31.92%)	
Hispanic	332 (10.77%)	322 (10.45%)	318 (10.31%)	
Other	60 (1.95%)	65 (2.11%)	48 (1.56%)	
Baseline blood pressure, mm Hg				
Systolic (mm Hg)	139.35 ± 15.38	139.06 ± 15.30	140.57 ± 16.05	<0.001
Diastolic (mm Hg)	77.73 ± 11.67	78.39 ± 11.71	78.25 ± 12.40	0.070
Distribution of systolic blood pressure, *n* (%)			0.030	
≤ 132 mm Hg	1050 (34.07%)	1076 (34.91%)	974 (31.59%)	
>132 to <145 mm Hg	1007 (32.67%)	994 (32.25%)	1001 (32.47%)	
≥145 mm Hg	1025 (33.26%)	1012 (32.84%)	1108 (35.94%)	
Serum creatinine, mg/dL	1.04 ± 0.32	1.07 ± 0.33	1.11 ± 0.36	<0.001
Urine albumin/creatinine ratio, mg/g Cr, median (Q1–Q3)	9.16 (5.50–19.40)	9.38 (5.63–20.59)	10.00 (5.77–24.01)	<0.001
Estimated GFR, mL min−1 1.73 m−2, median (Q1–Q3)	71.65 (59.16–84.47)	72.23 (58.90–85.16)	70.23 (56.18–84.21)	0.001
Fasting total cholesterol, mg/dL, median (Q1–Q3)	187 (161–215)	187 (162–214)	186 (160–215)	0.435
Fasting total triglycerides, mg/dL, median (Q1–Q3)	106 (75–148)	108 (77–152)	106 (78–150)	0.190
Fasting HDL cholesterol, mg/dL, median (Q1–Q3)	50 (43–61)	50 (43–60)	50 (42–60)	0.133
Fasting glucose, mg/dL, median (Q1–Q3)	97 (91–105)	97 (90–105)	97 (90–105)	0.432
Statin use, *n* (%)	1335 (43.54%)	1334 (43.55%)	1346 (44.02%)	0.913
Aspirin use, *n* (%)	1564 (50.76%)	1549 (50.39%)	1602 (52.10%)	0.371
Smoking status, *n* (%)			<0.001	
Never smoked	1445 (46.89%)	1353 (43.90%)	1274 (41.32%)	
Former smoker	1299 (42.15%)	1302 (42.25%)	1342 (43.53%)	
Current smoker	336 (10.90%)	422 (13.69%)	465 (15.08%)	
Framingham 10-y CVD risk score, %, median (Q1–Q3)	17.10 (11.72–24.79)	17.49 (11.64–25.68)	18.59 (12.73–26.42)	<0.001
No. of Antihypertensive agents	1.78 ± 1.02	1.81 ± 1.04	1.92 ± 1.05	<0.001
Not using antihypertensive agents, *n* (%)	305 (9.90%)	315 (10.22%)	252 (8.17%)	<0.001
Composite cardiovascular outcomes	127 (4.12%)	155 (5.03%)	271 (8.79%)	<0.001

### The Association Between Visit-to-Visit Heart Rate Variability and Composite Cardiovascular Outcomes

The association between visit-to-visit HRCV and composite cardiovascular outcomes was shown in Table 2. We used the low HRCV tertile as the reference. Participants with the high tertile had a significant higher risk of composite cardiovascular outcomes and this relation was still consistent after slight and full adjustment. As shown in fully adjusted Model 3, participants in the high HRCV tertile had the highest risk of outcomes [HR = 2.09, 95%CI (1.68, 2.59), *P* < 0.001]. The risk of composite cardiovascular outcomes in the middle HRCV tertile [HR =1.15, 95%CI (0.91, 1.47), *P* = 0.25] was not significantly different from that in the low HRCV tertile in Model 3. We also used HRCV as a continuous variable to investigate the relationship between HRCV and outcomes (Table 3). HRCV was significantly associated with increased risk of outcomes, even after adjusting for various covariates. Per 1 CV increment in HRCV, the hazard ratio of the risk of composite cardiovascular outcomes was 1.04 (95CI: 1.03, 1.05) in fully adjusted Model 3. The relationship between HRCV and outcomes was consistent between intensive and standard blood pressure control group. The relationship between visit-to-visit heart rate variability and composite cardiovascular outcomes was also consistent when heart rate variability was assessed by standard deviation of heart rate (Table 3).

**Table 2:** Association between visit-to-visit heart rate variability and composite cardiovascular outcomes in different models.

**VVHRV**	**Hazard ratio (95% CI)** ***P*****-value**		
	**Model 1**	**Model 2**	**Model 3**
**CV in heart rate**
T1	Ref.	Ref.	Ref.
T2	1.17 (0.93, 1.48) *P* = 0.18	1.20 (0.95, 1.52) *P* = 0.13	1.105 (0.91, 1.47) *P* = 0.25
T3	2.09 (1.70, 2.59) *P* < 0.001	2.09 (1.69, 2.58) *P* < 0.001	2.09 (1.68, 2.59) *P* < 0.001

*Model 1 adjusted for none*.

*Model 2 adjusted for age, sex, race and treatment arms*.

*Model 3 adjusted for age, sex, race, treatment arms, baseline systolic BP, baseline heart rate, smoking status, eGFR, serum creatinine, urine albumin/creatinine ratio, fasting triglycerides, Framingham 10-y CVD risk score, prior CVD and prior CKD*.

**Table 3:** Continuous Association between visit-to-visit heart rate variability and composite cardiovascular outcomes in different models.

**VVHRV**	**Hazard ratio (95%CI)** ***P*****-value**		
	**Model 1**	**Model 2**	**Model 3**
HRCV (per 1 CV increment)		*P* for interaction = 0.248	*P* for interaction = 0.390
Total	1.05 (1.04, 1.06) *P* < 0.001	1.04 (1.03, 1.05) *P* < 0.001	1.04 (1.03, 1.05) *P* < 0.001
Standard BP control	1.04 (1.02, 1.06) *P* < 0.001	1.03 (1.01, 1.05) *P* < 0.001	1.03 (1.01, 1.06) *P* < 0.001
Intensive BP control	1.05 (1.04, 1.07) *P* < 0.001	1.05 (1.03, 1.06) *P* < 0.001	1.04 (1.03, 1.06) *P* < 0.001

*Model 1 adjusted for none*.

*Model 2 adjusted for age, sex, race*.

*Model 3 adjusted for age, sex, race, baseline systolic BP, baseline heart rate, smoking status, eGFR, serum creatinine, urine albumin/creatinine ratio, fasting triglycerides, Framingham 10-y CVD risk score, prior CVD and prior CKD*.

**Figure 2:**
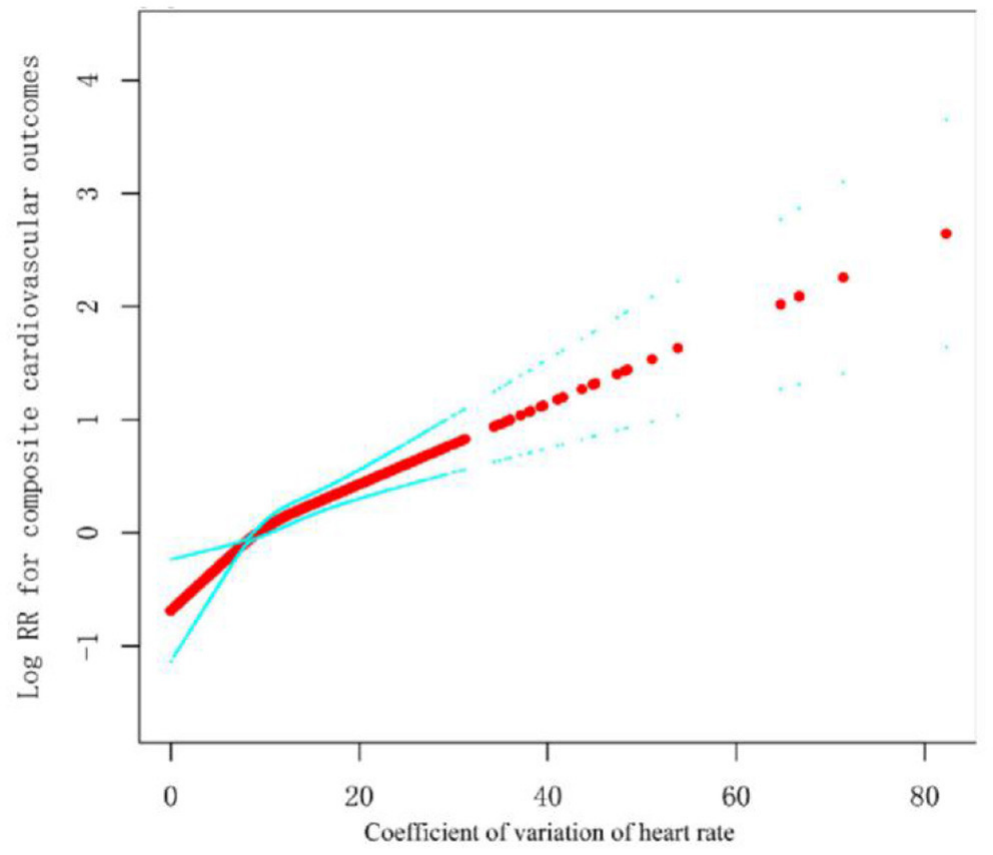
Smooth spline curves of visit-to-visit heart rate variability for the estimation of risk of composite cardiovascular outcomes. The red dot is Log HR, and the blue dot is 95%CI.All covariables in Model 3 were adjusted.

**Figure 3:**
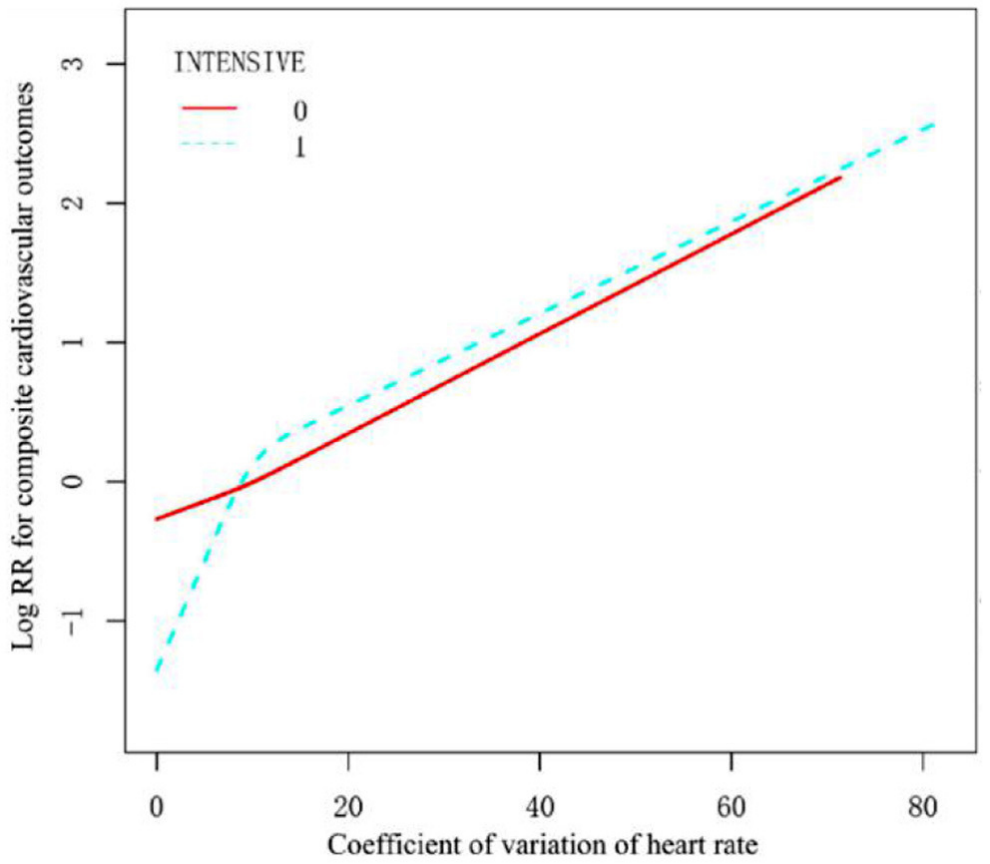
Smooth spline curves of visit-to-visit heart rate variability for the estimation of risk of composite cardiovascular outcomes stratified by treatment arms. The red solid line (INTENSIVE 0) is standard, and the blue dotted (INTENSIVE 1) line is intensive BP control. All covariables in Model 3 except treatment arms were adjusted.

The restricted cubic splines in Figure 2 showed that the continuous association between visit-to-visit heart rate variability (assessed by HRCV) and outcomes was liner. The relationship between them was consistent stratified by treatment arms (Figure 3). We also performed stratified analyses to assess the impact of HRCV (per 1 CV increment) on composite cardiovascular outcomes. The relationship between HRCV and outcomes were consistent in the prescribed subgroups. However, there was a significant interaction between age (<5 years vs. ≥75 years; *P* for interaction = 0.013) or systolic blood pressure categories (≤ 132 mmHg vs. 133–144 mmHg vs. ≥145 mmHg; *P* for interaction = 0.003) and HRCV on composite cardiovascular outcomes. The effect of HRCV on the risk of composite cardiovascular outcomes was smaller in participants aged 75 years or older [HR =1.03, 95%CI (1.01, 1.05), *P* = 0.004] than in those aged <75 years [HR =1.06, 95%CI (1.04, 1.08), *P* < 0.001]. HRCV remained significantly associated with increased risk of outcome in patients with baseline systolic blood pressure ≤ 132 mmHg or between 133 and 144 mmHg, but not in patients with baseline systolic blood pressure ≥145 mmHg [HR =1.02, 95%CI (0.99 1.04), *P* = 0.208]. The results in other subgroups were shown in Supplementary Figure S1.

### Visit-to-Visit Heart Rate Variability and Intensive Blood Pressure Control

**Figure 4:**
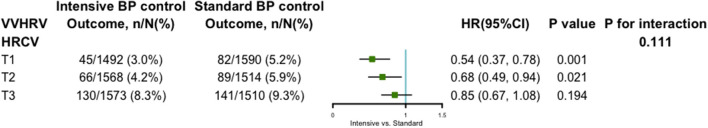
The impact of intensive vs. standard BP control on composite cardiovascular outcomes stratified by the tertiles of CV in heart rate. All covariables in Model 3 were adjusted.

Figure 4 showed the impact of intensive blood pressure control on the composite cardiovascular outcomes stratified by the HRCV tertiles. Intensive blood pressure control still had a significant reduction in the risk of outcomes in the low and middle but not high in the tertiles of HRCV. However, there was no significant interaction effect between HRCV (*P* for interaction = 0.111) and intensive blood pressure control.

## Discussion

In this study, we found a positive association between VVHRV and the risk of composite cardiovascular outcomes among the elderly with hypertension. Increased VVHRV was an independent predictor of a higher risk of cardiovascular events. The benefit of intensive blood pressure control in managing cardiovascular events was consistent in different VVHRV subgroups. In clinical work, long-term heart rate variability should be paid more attention rather than one-time measurement. VVHRV should be paid attention to in the management of elderly patients with hypertension, whether using standard or intensive blood pressure treatment.

The effect of long-term HR variability on health outcomes was studied in several epidemiology research. A population-based prospective cohort study in the Kailuan Chinese community found long-term RHR variability to be positively associated with all-cause mortality (7). Results from the Ohasama study among general Japanese population concluded that long-term variation of RHR was predictive of cardiovascular mortality (13). However, that study was limited by its self-measurement of VVHRV. Floyd et al. investigated the effect of variation in RHR over 4 years on the risk of myocardial infarction (MI) among older persons free of cardiovascular disease and found VVHRV being one of the most promising prognostic factors of MI, yet this study was limited by its small sample size and this finding was not extrapolated to other cardiovascular diseases (4). Compared with previous studies, our study has more advantages. First, our study has a large sample size and extremely high data quality. Second, we investigated for the first time whether VVHRV influences intensive blood pressure management. Consistent with previous studies, in our study, we found VVHRV, measured by HR-CV, was positively associated with composite cardiovascular outcomes in older patients with hypertension, and this result was consistent in different VVHRV subgroups. We also found the interaction between age or systolic blood pressure categories and HRCV on composite cardiovascular outcomes. This may be due to the higher Framingham 10-year CVD risk score and higher other cardiovascular risk factors among older adults and those with higher baseline blood pressure, which may influence the independent association between VVHRV and composite cardiovascular outcomes. These patients tended to have higher VVHRV, more complex disease conditions, and higher event rates (Supplementary Figure S5), and these confounding factors may attenuate the association between VVHRV and adverse events. This result was very similar to the original SPRINT study in that the benefit of intensive blood pressure management was relatively lowest in the group of patients with the highest systolic blood pressure,while intensive blood pressure management was not statistically significance in the group of patients with the highest systolic blood pressure.

The causal mechanism of long-term variation of RHR leading to cardiovascular outcomes remained unclear. It was hypothesized that VVHRV reflects sympathetic overactivity, which may induce myocardial work and activate platelet, leading to several cardiovascular outcomes including thrombosis and arrhythmia (14). VVHRV was also believed to be linked with neurocardiac functions relates to autonomic nervous system, such as stress, autonomic balance, vascular tone, and blood pressure, which was proven to be associated with risks of cardiovascular events (15).

Both RHR and VVHRV were proven to be positively associated with the risk of cardiovascular outcomes. A sub-study of the SPRINT randomized controlled trial found that the increase in the risk of composite cardiovascular events toward elevated baseline RHR was more potent in hypertensive subjects received intensive blood pressure treatment, compared to those with standard blood pressure treatment (10). However, the effect of intensive blood pressure control on VVHRV remained unknown. Our study showed VVHRV was positively associated with the risk of the composite cardiovascular outcomes in both intensive and standard blood pressure treatment arms. The benefit of intensive blood pressure control was consistent in VVHRV subgroups in hypertensive patients. Our findings provided suggestive evidence that VVHRV should be paid attention to in the management of elderly patients with hypertension, whether using standard or intensive blood pressure treatment.

## Conclusion

In conclusion, this *post-hoc* analysis of SPRINT trial found that long-term HR variability was positively associated with the risk of composite cardiovascular outcomes among older patients with hypertension. Intensive blood pressure control did not change the above association, and the benefits of intensive blood pressure management were consistent across different VVHRV groups. In clinical settings, less attention need be paid on VVHRV when treating hypertensive patients with intensive blood pressure regimen. Further studies were needed to extrapolate these results to the general population.

## Data Availability Statement

Publicly available datasets were analyzed in this study. This data can be found here: https://biolincc.nhlbi.nih.gov/studies/sprint/.

## Author Contributions

RZ and ZW completed the writing of the paper. KY applied for the database and made statistical analysis. WC was responsible for the revision of the paper. All authors confirmed the final version of the paper, contributed to the article, and approved the submitted version.

## Conflict of Interest

The authors declare that the research was conducted in the absence of any commercial or financial relationships that could be construed as a potential conflict of interest.

## Publisher's Note

All claims expressed in this article are solely those of the authors and do not necessarily represent those of their affiliated organizations, or those of the publisher, the editors and the reviewers. Any product that may be evaluated in this article, or claim that may be made by its manufacturer, is not guaranteed or endorsed by the publisher.
