# Supplementary material for: Visit-to-Visit Heart Rate Variability Is Positively Associated With the Risk of Adverse Cardiovascular Outcomes
Source: Front Cardiovasc Med. 2022 Mar 7;9:850223. doi: 10.3389/fcvm.2022.850223 (PMC8936423; doi:10.3389/fcvm.2022.850223)
Supplement: Supplementary file 1 [file Data_Sheet_1.docx]

Supplementary material

**Table S1.** Association between visit-to-visit heart rate variability(measured by HRSD) and composite cardiovascular outcomes in different models

| **VVHRV** | **Hazard ratio (95% CI) *P*-Value** | | |
| --- | --- | --- | --- |
|  | **Model 1** | **Model 2** | **Model 3** |
| SD in heart rate |  |  |  |
| T1 | Ref. | Ref. | Ref. |
| T2 | 1.04 (0.83, 1.30) P=0.72 | 1.13 (0.90, 1.41) P=0.30 | 1.11 (0.88, 1.40) P=0.37 |
| T3 | 1.57 (1.28, 1.92) P<0.001 | 1.68 (1.37, 2.06) P<0.001 | 1.67 (1.34, 2.07) P<0.001 |

Model 1 adjusted for none.

Model 2 adjusted for age, sex, race and treatment arms.

Model 3 adjusted for age, sex, race, treatment arms, baseline systolic BP, baseline heart rate, smoking status, eGFR, serum creatinine, urine albumin/creatinine ratio, fasting triglycerides, Framingham 10-y CVD risk score, prior CVD and prior CKD.

**Table S2.** Continuous Association between visit-to-visit heart rate variability(measured by HRSD) and composite cardiovascular outcomes in different models

| **VVHRV** | **Hazard ratio (95%CI) P-Value** | | |
| --- | --- | --- | --- |
|  | **Model 1** | **Model 2** | **Model 3** |
| **HRSD (per 1 SD increment)** |  |  |  |
| Total | 1.08 (1.05, 1.10) P<0.001 | 1.08 (1.06, 1.10) P<0.001 | 1.08 (1.06, 1.10) P<0.001 |
| Standard BP control | 1.05 (1.02, 1.09) P<0.001 | 1.06 (1.02, 1.09) P<0.001 | 1.06 (1.03, 1.09) P<0.001 |
| Intensive BP control | 1.11 (1.07, 1.14) P<0.001 | 1.11 (1.08, 1.14) P<0.001 | 1.11 (1.07, 1.14) P<0.001 |

Model 1 adjusted for none.

Model 2 adjusted for age, sex, race.

Model 3 adjusted for age, sex, race, baseline systolic BP, baseline heart rate, smoking status, eGFR, serum creatinine, urine albumin/creatinine ratio, fasting triglycerides, Framingham 10-y CVD risk score, prior CVD and prior CKD.

**Figure S1.** Smooth spline curves of visit-to-visit heart rate variability(measured by HRSD) for the estimation of risk of composite cardiovascular outcomes.


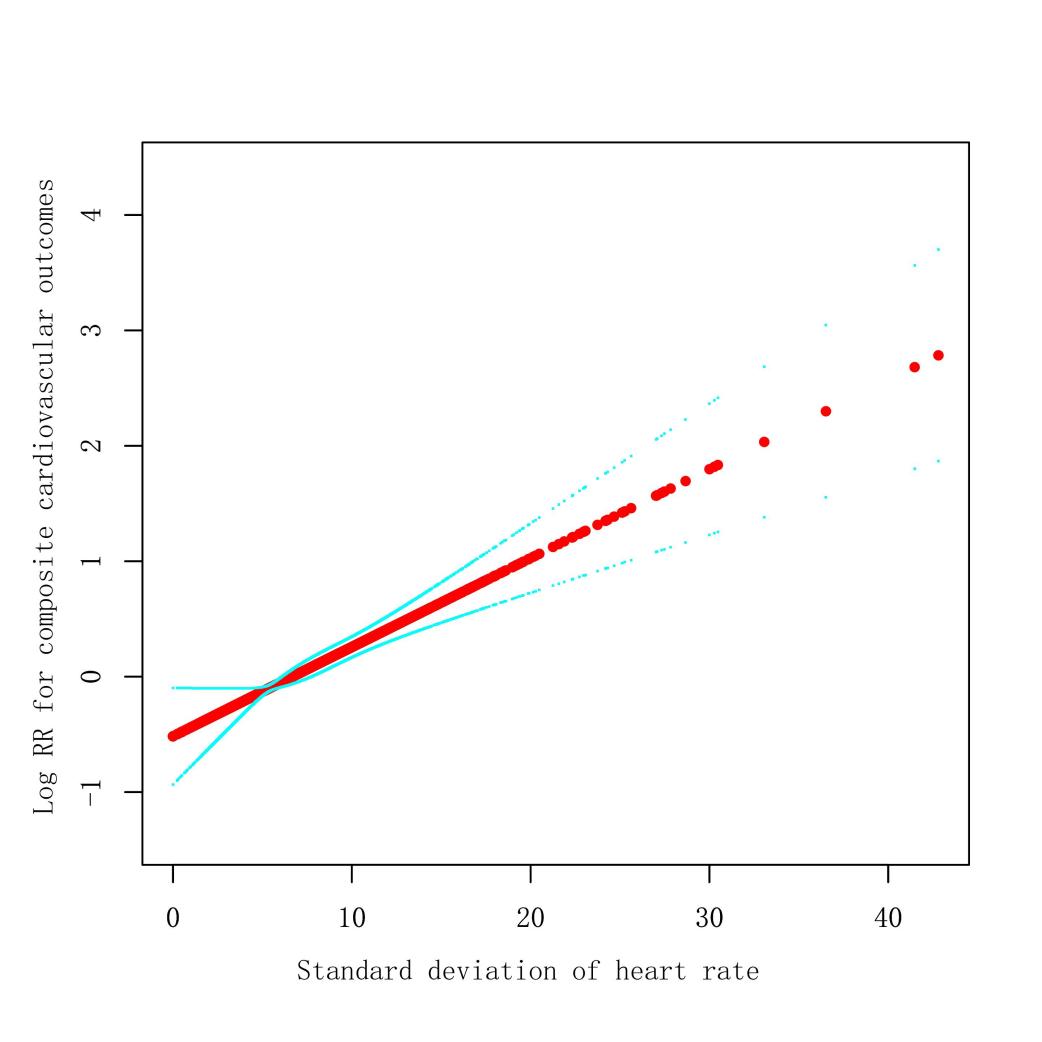


The red dot is Log HR, and the blue dot is 95%CI.

All covariables in Model 3 were adjusted.

**Figure S2**. Smooth spline curves of visit-to-visit heart rate variability(measured by HRSD) for the estimation of risk of composite cardiovascular outcomes stratified by treatment arms.
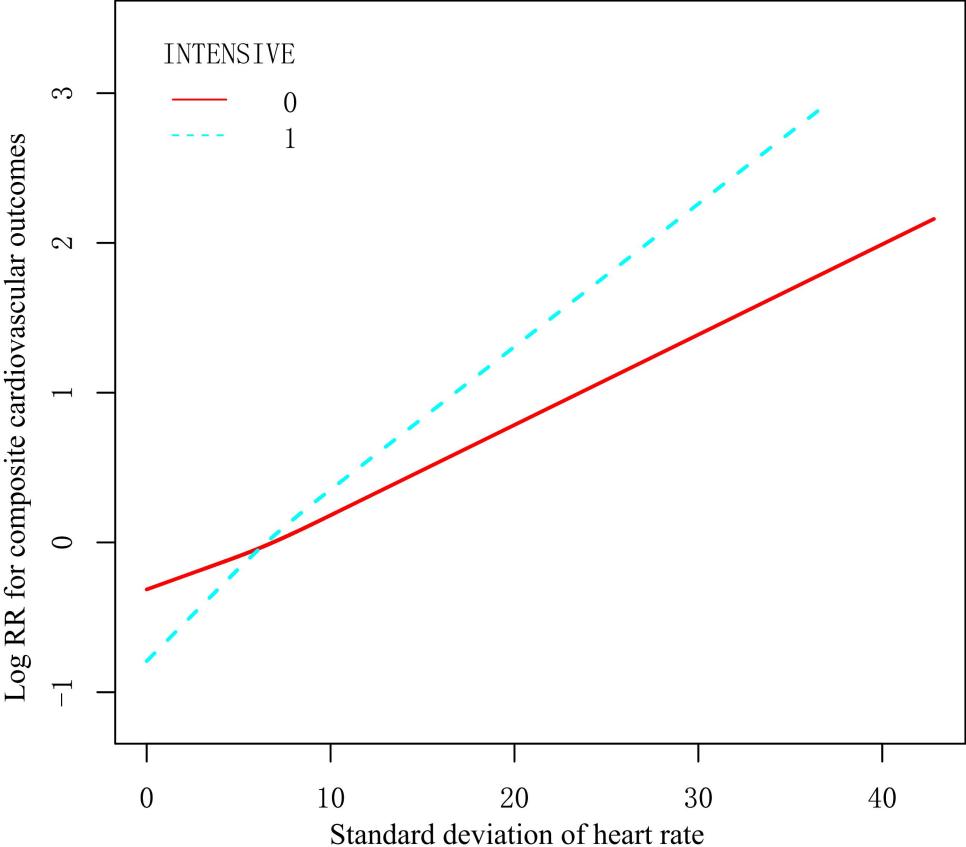


The red solid line(INTENSIVE 0) is standard, and the blue dotted(INTENSIVE 1) line is intensive BP control. All covariables in Model 3 except treatment arms were adjusted.

**Figure S3.** Subgroup analyses of the association between CV of HR and composite cardiovascular outcomes.
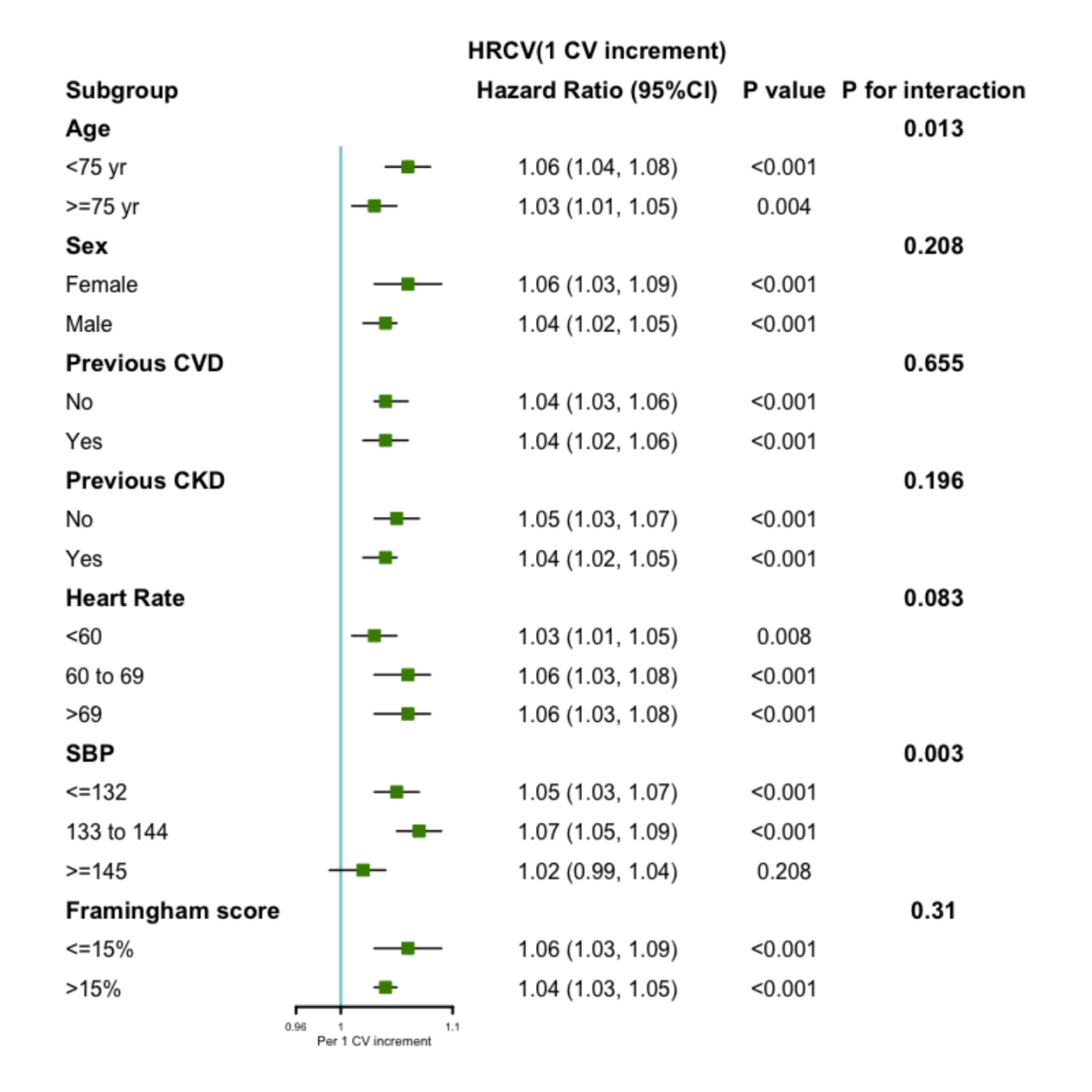


Each subgroup analysis was adjusted for all factors in Model 3, except for the stratification factor itself.

Figure S4. The impact of intensive versus standard BP control on composite cardiovascular outcomes stratified by the tertiles of HRSD.


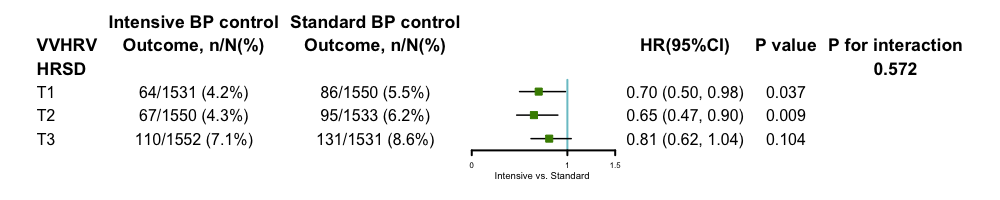


All covariables in Model 3 were adjusted.

Figure S5.


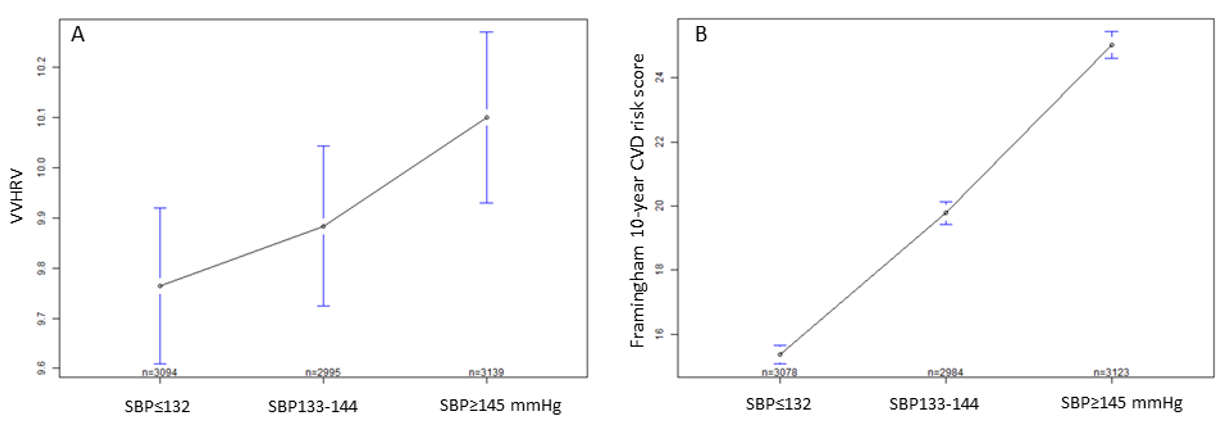


A.VVHRV in different SBP groups. B. Framingham 10-year CVD risk score in different SBP groups .
